# Association between maternal religious service attendance and pregnancy loss in the United States: a secondary analysis of the Future Families & Child Wellbeing Study

**DOI:** 10.21203/rs.3.rs-4913369/v1

**Published:** 2024-09-20

**Authors:** Stacie Shropshire, Andrew Williams

**Affiliations:** University of North Dakota, Grand Forks; University of North Dakota, Grand Forks

## Abstract

**Background.:**

Accumulating evidence suggests that spirituality and religiosity may be associated with improved health outcomes. However, few studies have examined maternal religiosity as a protective factor for perinatal outcomes. We explored the association between maternal religious attendance and pregnancy loss.

**Methods.:**

Data were drawn from the Future Families & Child Wellbeing Study’s first and second waves and medical records (n=1874). Religious attendance was a self-reported response to the question “About how often do you attend religious services?” Pregnancy loss was measured from responses to the second wave survey question, “Since focal child’s birth, have you had any miscarriages/abortions/stillbirths?” Logistic regression estimated odds ratios (OR) and 95% confidence intervals (CI) for the association between maternal religious attendance frequency and pregnancy loss, overall and by race. Models were adjusted for sampling weights, religious preference, socioeconomic and behavioral factors.

**Results.:**

8% (n=164) of mothers reported having a pregnancy loss. Of those with a pregnancy loss, 28% (n=46) attended services hardly ever and 20% (n=20) attended services once a week or more. Women who attended services more frequently had 58% increased odds of not experiencing a pregnancy loss (OR:1.58;95%CI:1.01,2.48) after adjusting for potential confounding. A post hoc analysis found no difference in pregnancy loss type or subsequent reproductive history based on attendance level.

**Conclusions.:**

Results suggest that higher maternal religious attendance frequency may be a protective factor for pregnancy loss. Further research is needed to understand the association between maternal religious attendance and mechanisms for pregnancy loss.

## 1. Introduction

### 1.1 Background Rationale

The United States has some of the highest maternal morbidity and mortality rates and adverse infant outcomes among developed nations and rates of adverse perinatal outcomes are steadily increasing within the U.S. [1–3]. Pregnancy loss, such as miscarriage and stillbirth, is the most common adverse perinatal outcome in the U.S. with approximately 750,000–1,000,000 miscarriages [4] and 21,000 stillbirths occurring annually [5]. Experiencing a pregnancy loss can result in negative physical and mental health for the birthing person and their respective partner [4]. While there is robust research evaluating coping methods for pregnancy loss, data are sparse regarding potential protective factors to reduce pregnancy loss and improve infant and maternal health outcomes [6, 7].

Within the United States, 45% of individuals consider religion a very important part of their life [8] and 21% report attending religious services every week [9]. Religious involvement may improve health through communal aspects such as fostering a sense of purpose and promoting healthy behaviors [10–12]. Recent research suggests religious involvement may be associated with improved health outcomes including reduced all-cause mortality risk [11], lower levels of chronic pain [13], lower prevalence of hypertension [14, 15], and fewer hospitalizations [16]. Specific to perinatal outcomes, three studies have investigated the association between maternal religious involvement and adverse perinatal health outcomes [17–19]. Religious service attendance was found to be a protective factor for low birth weight (LBW) in a cross-sectional study of mostly disadvantaged non-married women in the United States (n = 3583) with women attending services once a week or more having 80% reduced odds of LBW compared to women who reported no attendance [17]. A separate cross-sectional study of mainly disadvantaged and racially diverse women in the United States (n = 4166) found that mothers attending religious services once a week or more had 1.5 times greater odds of initiating breastfeeding than mothers with no attendance [18]. A prospective cohort study among American women receiving care in South Carolina and Mississippi (n = 307) found that religious participation of attending religious services at least a few times a month was protective of postpartum depression (PPD) [19]. However, religion is not monotonically beneficial to health, as religiosity may negatively influence mental health through promotion of “guilt, anxiety, violence, and intolerance” [11]. As the burgeoning evidence suggests religiosity is associated with healthy maternal and infant outcomes, examining the role of religiosity in pregnancy loss is warranted.

### 1.2 Objectives

To the best of our knowledge, there are no previous studies evaluating the association between maternal religiosity and pregnancy loss. Based on previous literature, we developed the following hypothesis: higher maternal religious attendance frequency is associated with increased odds of not experiencing a pregnancy loss.

## 2 Materials and Methods

### 2.1 Data Description

This study utilizes a cross-sectional design to examine Future of Families and Child Wellbeing Study (FFCWS) data. The FFCWS is a longitudinal cohort study conducted between 1998 and 2017 that follows 4898 live births from 75 hospitals in 20 U.S. cities with populations of 200,000 or more [20, 21]. Samples from each hospital were randomly selected until preset quotas were met to be representative of non-marital births in each city [21]. Baseline survey responses were collected from both mother and father shortly after the focal child’s birth between 1998 and 2000. Medical records were obtained at baseline for mother and child for 3684 births in the cohort with missing data occurring due to missing records (29%), refused consent (33%), too few cases to collect data or the hospital refused researchers access to records (38%). Follow-up waves were conducted when the focal child was aged 1, 3, 5, 9, and 15 years [20].

### 2.2 Sample

The sample was drawn from the mother baseline survey (Wave 1), mother medical records collected at baseline, and Year 1 follow-up mother survey (Wave 2). Inclusion criteria included participants who had data corresponding to religious service attendance frequency, religious preference, pregnancy loss and confounding variables. Exclusion criteria included missing data of interested variables. The sample size started at 4898 and was reduced to 3684 with inclusion of the medical record data. The final sample study size consists of 1874 mothers after excluding missing observations for variables of interest.

### 2.3 Outcome Variables

Pregnancy loss was measured using the response to the FFCWS second wave survey question, “Since focal child’s birth, have you had any miscarriages/abortions/stillbirths?” (0 = No, 1= Yes). For the main analyses, we included miscarriages, abortions and stillbirths in the pregnancy loss variable. This decision was made as there is no data regarding reasons for an abortion (e.g. elective vs medically indicated), and to preserve statistical power.

### 2.4 Exposure Variable

Maternal religious service attendance was measured using responses to the baseline survey question, “About how often do you attend religious services?” (1 = No attendance, 2 = Hardly ever, 3 = Several times a year, 4 = Several times a month, 5 = Once a week or more). For the inferential analyses, maternal attendance was considered a continuous variable to preserve statistical power.

### 2.5 Confounders

Confounders to account for socioeconomic status (SES) and demographic factors were informed by literature [17, 18, 22]: marital status (married, cohabitating, single), education (high school or less, some college or more), immigration status (immigrant, born in the U.S.), poverty level (< = 200% of poverty line, > 200% of poverty line), health insurance (no insurance, Medicaid/other, private insurance), and race/ethnicity (Non-Hispanic White, Non-Hispanic Black, Hispanic/Other). Other potential confounding variables that were accounted for include: general health status (fair/poor, good), substance use during pregnancy (no, yes), parity (one child, more than one child), early prenatal care initiation (yes, no), focal baby’s sex at birth (male, female), maternal age in years, and maternal pre-pregnancy body mass index (BMI) [23, 24]. As level of service attendance has been found to vary based on the influence of different religious traditions [10], religious preference was used as a control variable. Religious preference was measured using responses to the maternal baseline survey and recoded based on previous literature [17] and statistical power (0 = No Religion, 1 = Conservative Protestant, 2 = Other Christian, 3 = Catholic, 4 = Other Religion).

### 2.6 Analysis

Descriptive and inferential statistics were conducted utilizing SAS version 9.4. For descriptive statistics, unweighted frequencies and percentages were calculated for categorical variables while medians and interquartile ranges (IQR) were calculated for continuous variables. A series of weighted logistic regression models were fit to estimate odds ratios (OR) and 95% confidence intervals (CI) for the association between religious service attendance and pregnancy loss. Model 1 is adjusted for maternal religious preference. Next, marriage status, education, immigrant status, poverty level, and health insurance status were added in Model 2. General health status, substance use during pregnancy, parity, early prenatal care initiation, focal baby’s sex at birth, maternal age, and maternal pre-pregnancy BMI were added in Model 3. The fully adjusted model (Model 4) includes race/ethnicity.

In post hoc analysis we used Fischer’s exact test to explore associations between type of pregnancy loss (abortion, stillbirth/miscarriage) and religious service attendance. As those who frequently attend religious services are more opposed to abortion, this post hoc analysis may provide evidence that type of pregnancy loss varies across levels of religious attendance, potentially biasing our results [25]. Type of pregnancy loss was measured from the responses to the question, “Did you have a miscarriage (or stillbirth), an abortion, or both a miscarriage and abortion?” (1 = Miscarriage/Stillbirth, 2 = Abortion, 3 = Both).

In a second post hoc analysis, we used Pearson’s chi-square test to explore associations between pregnancy loss and reproductive history subsequent to the focal child’s birth. Reproductive history since focal birth was self-reported at the 1-year follow up via the question “Since focal child was born, have you had another baby or are you pregnant now?” and categorized as “had a subsequent birth/currently pregnant at the 1-year follow – up, no subsequent birth/not pregnant at the 1-year follow-up.” This analysis may provide information on pregnancy loss in the context of other reproduction-related events.

## 3 Results

### 3.1 Descriptive Statistics

The sample is made up of primarily unmarried cohabitating (n = 731, 39%) Non-Hispanic Black women (n = 719, 38%) in their 20s (median = 23; IQR: 20,29). The majority have a high school or less education (n = 1176, 63%), have more than one child (n = 1118, 60%), and were born in the United States (n = 1609, 86%) (Table 1). 58% (n = 1086) are under or equal to 200% of the poverty line and 54% (n = 1014) have Medicaid or other non/private health insurance. 27% (n = 503) of mothers reported attending religious services hardly ever, 14% (n = 258) reported no attendance, and 21% (n = 394) reported attending services once a week or more. The most prominent religious preference among the sample was Catholicism at 33% (n = 618) followed by Conservative Protestantism (n = 493; 26%). 9% (n = 164) of mothers reported having had a miscarriage, abortion, or stillbirth (Table 1).

The unweighted number and percentages for pregnancy loss by maternal attendance frequency can be seen in Table 2. Of those with a pregnancy loss, 28% (n = 46) attended services hardly ever and 20% (n = 20) attended services once a week or more (*P* = 0.016).

### 3.2 Inferential Statistics

Table 3 includes results from the series of logistic regression models. Model 1, adjusted only for religious preference, mothers who attended services more frequently had 63% increased odds (OR:1.63; 95% CI:1.11,2.39) of not experiencing a pregnancy loss. The association between maternal attendance and pregnancy loss remained after adjusting for religious preference, SES, behavioral and other factors, and race, with mothers who attended more frequently having 58% increased odds (OR:1.58; 95% CI:1.01,2.48) of not experiencing a pregnancy loss.

For the first post-doc analysis examining the association of pregnancy loss type by religious attendance, no difference was found between the types of pregnancy loss reported and the different levels of religious attendance (*P* = 0.89; Table 4).

For the second post-hoc analysis of the association between pregnancy loss and reproductive history subsequent to the focal birth, there was no difference in pregnancy loss by subsequent births of the focal child’s birth or currently pregnant at the 1-year follow up (Table 5).

## 4 Discussion

In this cross-sectional study of mainly unmarried U.S. mothers, we found a strong positive association between frequent maternal religious service attendance and not experiencing pregnancy loss that persisted after adjusting for religious preference, SES, behavioral, race/ethnicity, and other variables. These findings support our hypothesis that higher maternal religious attendance frequency is associated with increased odds of not experiencing a pregnancy loss. Results of this study add to the evidence that religious attendance may be a protective factor for adverse health outcomes and extend the literature by examining specifically the association between maternal attendance and pregnancy loss.

Though there are few studies regarding religious attendance and perinatal outcomes, our results align with prior work that has been conducted on this topic [17–19]. A separate examination of FFCWS data found that those who attended services once a week or more had 75% reduced odds of LBW compared to mothers who did not attend services [17]. For every one unit increase in attendance frequency, the odds of LBW were reduced by 15% (OR:0.85) after adjusting for confounding and addressing potential mediating [17]. Similarly, among a sample of mostly disadvantaged families, attending religious services once a week or more had a 49% (OR:1.49) increase in odds of initiating breastfeeding in comparison to not attending services [18]. A prospective cohort study found participation in organized religious activities of at least a few times a month reduced the odds of PPD by 82% (OR: 0.18) among a sample of mainly university educated married women [19].

It has been suggested that high religious attendance frequency may act as a protective factor for wellbeing due to promotion of healthy habits, increased social connections, and transcendent purpose and meaning [10, 26]. However, mechanisms linking religious attendance and pregnancy loss have not been previously described. We hypothesize that stress experienced during the perinatal period may influence biological functions which can subsequently increase the risk of pregnancy loss. Increased stress during pregnancy can elevate maternal cortisol levels (a stress-related hormone) which have been found to result in a higher risk of early pregnancy loss [27]. Evidence also suggests that high levels of stress during pregnancy are associated with altered placental function [28, 29]. Effects of high levels of stress and placental function may occur due to excessive levels of stress-related hormones [30]. As placental function is a key factor in pregnancy loss [31, 32], reducing or mitigating stress may decrease the risk of pregnancy loss. Frequent religious attendance may mitigate these effects, as religious people may have lower levels of cortisol compared to individuals who identify as agnostic, spiritual, or atheist [33, 34]. Thus, the mechanism of religious attendance mitigating adverse effects of stress during the perinatal period is feasible, however, further investigation is warranted.

There may be certain aspects of religious attendance that reduce stress or buffer its effects such as the idea of forgiveness. Existing evidence supports the association between practicing forgiveness and reduced stress and anxiety [26, 35]. Mothers who attend services more frequently may be more likely to practice the idea of forgiveness, which can potentially lead to lower levels of stress and thus, reduced risk of pregnancy loss. Attending religious services more frequently can also lead to greater life satisfaction, optimism, and hope [26]. Additionally, mothers with high attendance may have stronger social connections with members in their respective church communities and more access to social services that are often provided by religious organizations than those who do not attend services or those who attend less regularly [26]. The strength of the social connections in relation to religious attendance may be stronger than that of other social involvement due to regular and repeated social contact with community members who share similar deep beliefs and values [26]. Combined, these influences may lead to improved psychosocial and psychological health which can potentially reduce stress by improving biological function and thus, lower risk of pregnancy loss. Further research is needed to identify stressors that may be reduced by frequent religious attendance to better understand potential mechanisms that protect against pregnancy loss. Future studies may also focus on the social aspect of religious attendance and its respective strength in comparison to other organizations and community groups.

Though our results have found maternal religious attendance to be a protective factor for pregnancy loss, they do not imply that religious attendance should be considered as a prescription or treatment [36]. Rather, providers may choose to encourage religiously inclined patients to engage with their respective faith community when appropriate. For patients who are non-religious, it may be appropriate at times to encourage exploring mediating factors such as what gives their life meaning or social connections [36].

### 4.1 Limitations

There are a few limitations to this current study. First, the FFCWS sample is primarily births to disadvantaged mothers in large urban areas, potentially limiting generalizability of our results. Second, due to sample variance and low statistical power, religious attendance was used as a continuous variable within the logistic regression models and thus, differences may exist between the levels of attendance. Still, we were still able to assess the linear aspect of religious attendance and determine that a higher frequency of attendance was protective of pregnancy loss. Third, as pregnancy loss was self-reported, it is possible that recall bias may have been introduced; however, recall of events surrounding pregnancy is generally reliable [37, 38].

### 4.2 Strengths

There are several notable strengths of the study. To the best of our knowledge, this study is the first to examine religiosity and pregnancy loss. As this study contains an over-sampled high-risk population of mothers, our findings suggest that religiosity and components of religious involvement such as reduced stress, social connections, and life meaning may be positive influences regarding perinatal health among a high-risk group. In addition, many sociodemographic variables were able to be included in the analysis including religious preference. Accounting for religious preference allows for conclusions to be made between religious attendance and pregnancy loss regardless of religiosity patterns of respective faith groups. Thus, our findings are more cross-cutting. Thorough consideration of potential confounders was completed to include the most important risk factors as identified by previous literature.

It is notable that our post-hoc analyses suggest there is no variation in type of pregnancy loss by religious attendance, nor variation in experiencing pregnancy loss by reproductive history subsequent to the focal birth. Thus, in this sample, women with high religious attendance are not less likely to report an abortion compared to women with low religious attendance. Additionally, women with confirmed pregnancies since the focal child’s birth are not more likely to report pregnancy loss than women not currently pregnant. Given these findings, our concerns regarding biased estimates are minimal.

## 5 Conclusion

Pregnancy loss remains a persistent adverse perinatal outcome in the United States. This cross-sectional study of secondary data from the FFCWS found an association between increased maternal religious attendance and reduced odds of pregnancy loss after adjusting for potential confounding. We suggest that frequent attendance may protect against pregnancy loss by mitigating against increased stress levels during the perinatal period. Multiple components of increased religious attendance including social connections, beliefs, and practices may reduce stress thus protecting against high levels of cortisol and altered placental function during pregnancy leading to reduced pregnancy loss. Further research is warranted to investigate this potential mechanism pathway.

## Figures and Tables

**Table 1:** Descriptive statistics of analytic sample (n = 1874)

Variables	Unweighted number (%)
Sample Size = 1874	
*Independent Variable*	
**Religious Service Attendance**	
No attendance	258 (13.77)
Hardly ever	503 (26.84)
Several times a year	395 (21.08)
Several times a month	324 (17.29)
Once a week or more	394 (21.02)
**Dependent Variables**	
**Loss of Pregnancy**	
No miscarriages/abortions/stillbirths	1710 (91.25)
Had a miscarriage/abortion/stillbirth	164 (8.75)
**Socio-demographic controls**	
**Religious Preference**	
No Religion	210 (11.21)
Conservative Protestant	493 (26.31)
Other Christian	363 (19.37)
Catholic	618 (32.98)
Other Religion	190 (10.14)
**Marriage Status**	
Married	450 (24.01)
Cohabitating (Unmarried)	731 (39.01)
Single	693 (36.98)
**Education**	
High school or less	1176 (62.75)
Some college or more	698 (37.25)
**Immigration Status**	
Immigrant	265 (14.14)
Born in the U.S.	1609 (85.86)
**Poverty Level**	
<= 200% of poverty line	1086 (57.95)
> 200% of poverty line	788 (42.05)
**Health Insurance**	
No insurance	220 (11.74)
Medicaid/other	1014 (54.11)
Private insurance	640 (34.15)
**General Health Status**	
Fair/poor	117 (6.24)
Good	1757 (93.76)
**Substance Use During Pregnancy**	
No	1393 (74.33)
Yes	481 (25.67)
**Parity**	
1 child	756 (40.34)
More than 1 child	1118 (59.66)
**Early Prenatal Care Initiation**	
Yes	1513 (80.74)
No	361 (19.26)
**Focal Baby’s Sex at Birth**	
Male	993 (52.99)
Female	881 (47.01)
**Race/Ethnicity**	
Non-Hispanic White	496 (26.47)
Non-Hispanic Black	719 (38.37)
Hispanic / Other	659 (35.17)
Variables	Median (IQR)
**Maternal Age in Years**	23.00 (20.00, 29.00)
**Maternal Pre-Pregnancy BMI**	24.69 (21.22, 29.76)

Note. IQR = Interquartile Range

**Table 2:** Pregnancy loss by maternal religious service attendance descriptive statistics (n = 1874)

Variables	No Loss of Pregnancy *Unweighted # (Column %)*	Loss of Pregnancy *Unweighted # (Column %)*
No attendance	228 (13.33)	30 (18.29)
Hardly ever	457 (26.73)	46 (28.05)
Several times a year	352 (20.58)	43 (26.22)
Several times a month	299 (17.49)	25 (15.24)
Once a week or more	374 (21.87)	20 (12.20)
Chi-square *P*-value	0.0164	

**Table 3:** Association between frequency of maternal religious service attendance & pregnancy loss (n = 1874)

Variables	Model 1 *OR (95% CI)*	Model 2 *OR (95% Cl)*	Model 3 *OR (95% Cl)*	Model 4 *OR (95% Cl)*
Maternal Religious Service Attendance Frequency	1.63 (1.11, 2.39) *	1.59 (0.92, 2.73)	1.58 (1.01, 2.46) *	1.58 (1.01, 2.48) *
**Religious Preference**				
Other Religion	1.62 (0.30, 8.79)	1.30 (0.23, 7.45)	1.20 (0.22, 6.59)	1.20 (0.22, 6.62)
Catholic	0.66 (0.10, 4.81)	0.80 (0.19, 3.35)	0.83 (0.21, 3.34)	0.81 (0.14, 4.67)
Other Christian	2.43 (0.79, 7.46)	2.06 (0.69, 6.15)	2.50 (0.71, 8.81)	2.51 (0.67, 9.39)
Conservative Protestant	0.89 (0.32, 2.46)	0.92 (0.30, 2.81)	0.94 (0.31, 2.83)	0.95 (0.29, 3.14)

Note. CI = Confidence Interval;

* p < 0.05.

**Table 4:** Pregnancy loss type by maternal religious service attendance (n = 163)

Variables	Miscarriage/Stillbirth *Unweighted # (Column %)*	Abortion *Unweighted # (Column %)*	Both *Unweighted # (Column %)*
No attendance	16 (20.51)	12 (16.44)	2 (16.67)
Hardly ever	19 (24.36)	24 (32.88)	3 (25.00)
Several times a year	21 (26.92)	19 (26.03)	2 (16.67)
Several times a month	11 (14.10)	11 (15.07)	3 (25.00)
Once a week or more	11 (14.10)	7 (9.59)	2 (16.67)
Fischer’s Exact *P*-value	0.89		

**Table 5:** Pregnancy loss by subsequent reproductive history (n = 1871)

Variables	No Loss of Pregnancy *Unweighted # (Column %)*	Loss of Pregnancy *Unweighted # (Column %)*
Subsequent Birth/Currently Pregnant	221 (11.81)	25 (15.24)
No Subsequent Birth & Not Currently Pregnant	1486 (79.42)	139 (84.76)
Chi-square *P*-value	0.41	

## Data Availability

The data that support the findings of this study are available from FFCWS, but restrictions apply to the availability of these data, which were used under a Contract Data Agreement for the current study, and so are not publicly available. Data are however available from the authors upon reasonable request and with permission of FFCWS.
